# IGPred-HDnet: Prediction of Immunoglobulin Proteins Using Graphical Features and the Hierarchal Deep Learning-Based Approach

**DOI:** 10.1155/2023/2465414

**Published:** 2023-01-25

**Authors:** Zakir Ali, Fahad Alturise, Tamim Alkhalifah, Yaser Daanial Khan

**Affiliations:** ^1^Department of Computer Science, School of Science and Technology, University of Management and Technology, Lahore, Pakistan; ^2^Department of Computer, College of Science and Arts in Ar Rass, Qassim University, Ar Rass, Qassim, Saudi Arabia

## Abstract

*Motivation*. Immunoglobulin proteins (IGP) (also called antibodies) are glycoproteins that act as B-cell receptors against external or internal antigens like viruses and bacteria. IGPs play a significant role in diverse cellular processes ranging from adhesion to cell recognition. IGP identifications via the in-silico approach are faster and more cost-effective than wet-lab technological methods. *Methods*. In this study, we developed an intelligent theoretical deep learning framework, “IGPred-HDnet” for the discrimination of IGPs and non-IGPs. Three types of promising descriptors are feature extraction based on graphical and statistical features (FEGS), amphiphilic pseudo-amino acid composition (Amp-PseAAC), and dipeptide composition (DPC) to extract the graphical, physicochemical, and sequential features. Next, the extracted attributes are evaluated through machine learning, i.e., decision tree (DT), support vector machine (SVM), k-nearest neighbour (KNN), and hierarchical deep network (HDnet) classifiers. The proposed predictor IGPred-HDnet was trained and tested using a 10-fold cross-validation and independent test. *Results and Conclusion*. The success rates in terms of accuracy (ACC) and Matthew's correlation coefficient (MCC) of IGPred-HDnet on training and independent dataset (D_train_ D_test_) are ACC = 98.00%, 99.10%, and MCC = 0.958, and 0.980 points, respectively. The empirical outcomes demonstrate that the IGPred-HDnet model efficacy on both datasets using the novel FEGS feature and HDnet algorithm achieved superior predictions to other existing computational models. We hope this research will provide great insights into the large-scale identification of IGPs and pharmaceutical companies in new drug design.

## 1. Introduction

Immunoglobulins are serum proteins in the human body. These proteins act as an antibody involved in the various cellular processes such as a decision, binding, or recognition of the cell. Immunoglobulin significantly boosts the immune system by discovering the dangerous macromolecules that entered the body [1]. When unfamiliar elements inject into the body, the immune system has a unique skill to detect the attacker and then activates B lymphocytes to hide the immunoglobulin from invader antigens. For instance, immunoglobulins will deactivate the toxin by altering its chemical structure when averting its appearance. To provide a shield against bacterial infection, stabilin-2 can attach to both Gram-positive and Gram-negative bacterial contagions.

Immunoglobulins are linked/related to various disease treatments [2], such as autoimmune, inflammation in the skin, and Bechet's diseases [3, 4]. In other words, intravenous immunoglobulin provides a fighting strength to cure such kinds of diseases for people who have suffered from muscle problems and systemic swelling in skin infections. The use of immunoglobulin for lupus erythematosus dermatosis in association with the treatment of Bechet's infection has a great potential without any harmful impact [3, 4]. In Ref. [5], it is shown that immunoglobulins have a better understanding of immunological processes, permitting the development of an enhanced version of drugs to cure the infection. Considering the medical application of immunoglobulin proteins, in-depth knowledge of their functional level is still under development.

Over the past years, immunoglobulin protein classification and characterization have become a hot topic in bioinformatics and computational biology. Wet-lab approaches such as X-ray crystallography and mass spectrometry are used to discover immunoglobulin proteins. However, such laboratory-based approaches are unfavourable due to their high cost and time consumption. In this regard, researchers have designed various machine learning-based methods to identify immunoglobulin protein sequence analysis. Efficient machine learning-based methods can quickly and accurately predict unannotated proteins from large databases. Machine learning techniques are applied in numerous areas of medicine like diagnostics. Clonal dynamics and relative frequencies are utilized to develop an antibody clonal examining framework to explore certain antigenic human monoclonal antibodies [5–7]. In the various field of the healthcare system, immunological and biological usage, including infection control, immunization diagnostics, and B-cell detection, is of key significance [8, 9]. The research community has reported numerous studies related to antigen range that can be selected by specific antibodies or by a group of antibodies, e.g., antibody stock provided by applying a Rep-Seq in many areas [10]. The said key observation headed to another and well-defined technique for tackling the B-cell epitope detection in which the intellectual purpose of a specific antibody is detected [11, 12]. This study incorporates optical, electrochemical, and piezoelectric biosensors to predict complete immunoglobulin degrees, in which electrochemical is most generally employed. Several immunoglobulin optical biosensors depend on surface plasmon resonance (SPR) prediction present in buffer solutions. For an immunoglobulin study, these available state-of-the-art technologies are useful; however, conducting the biochemical study is very expensive in terms of money and time. For accurate and speedy execution of a huge amount of protein data, it is a need of time to develop a computational framework for immunoglobulins. For example, the first phase declares the purpose of immunoglobulins proteins which design a useful and inexpensive framework to predict them efficiently. The research community has designed various frameworks based on machine learning procedures for protein sequence analysis and classification in the last decades [13–17]. In bioinformatics, predicting immunoglobulins transforms protein sequences into feature metrics to uncover the core formation of proteins. The essential characteristics of protein prediction are itemized as follows: feature representation and key feature selection based on their importance and classification. Amino acid composition (AAC), dipeptides (Dip), and tripeptides are feature extraction techniques to extract *n*-gram features representation, where the occurrence of *n*-length peptides are utilized as feature matrices [18–20].

Furthermore, another feature extraction method pseudo-amino acid composition (PseAAC), is commonly implemented, considering physicochemical properties among residues [15, 17, 21–23]. The pseudotype protein structure led to a protein density drop in dyscalculia; for this purpose, the notion of pseudo-K-tuple is combined with the idea of PseAAC [24, 25] to design a framework of AAC minimized with pseudo-K-tuples amino acid composition (PseKRAAC) [26]. They developed a classifier IGPred by considering nine (9) physicochemical properties of amino acid-generated proteins with replica ACC [27, 28]. In Ref. [29], a predictor was developed via a support vector machine (SVM) to predict immunoglobulins and nonimmunoglobulins. They used PseAAC with nine physical and chemical characteristics of amino acids; A cross-validation technique was used to train a model, and they got 96.3% accuracy. However, the performance is good but still needs an efficient bioinformatics tool to predict immunoglobulin with a less error rate.

Various feature representations and multifaced prediction methods may produce unnecessary knowledge representation [30, 31]. However, to deal with this problem, many studies suggested feature selection algorithms for eliminating unnecessary information to enhance the performance of the prediction methods. The first one is PCC, which stands for Pearson's correlation coefficient, used to measure the significance of feature representation in a subgroup. In contrast, the second part is related to computing the repetition among features representation by using Euclidean distance (ED), cosine distance (CD), and Tanimoto (TO). Maximum-Relevance-Maximum-Distance in [32, 33] and Analysis of Variance (ANOVA) in [34] are typical feature selection approaches. For optimum feature representation, [35–37] used the principal component analysis (PCA) and misclassification error (MCE) to extract optimal feature representation for pentatricopeptide-repeat proteins prediction and got 97.9% accuracy. Li et al. in [33] used the above method to design a model for the prediction of anticancer peptide sequences with 19-dimensional attributes.

Although significant contribution has been devoted to the prediction of IGPs, some shortcomings should be acknowledged in terms of feature-encoding schemes and learning models. One major limitation of the existing methods is the lack of feature learning algorithms to extract the structured pattern information from protein sequences properly. Secondly, only machine learning classifiers are not accurate enough to discriminate IGPs from non-IGPs. Thirdly, the developed immunoglobulin predictors only showed the training dataset results using a cross-validation test while ignoring the external/independent test results. Independent test results are significant as they show the trained model's generalization power.

To our best knowledge, IGPred-HDnet is the first deep learning-based predictor for identifying IGPs. IGPred-HDnet extracts the nominal feature vectors using novel feature descriptors such as FEGS (extract the graphical features), AAPse (extracting physicochemical features), and DPC (sequential features) from the given protein sequence and fed to the hierarchical deep net model (HDnet) as the base classifier for constructing the model. The model opts for deep representations instead of manually extracted handcrafted features and aims to perform the classification of IGPs. We have validated the model through exhaustive methods which shows that the overall prediction on both training and testing datasets outperformed the existing state-of-the-art methods. The study provides great insights into the large-scale identification of IGPs which pharmaceutical companies can opt for in novel drug design.

## 2. Materials and Methods

**Figure 1:**
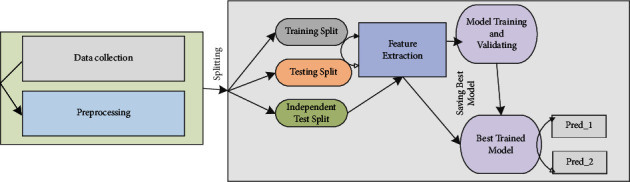
Stepwise approaches for classification of IGPs.

In the subsequent subsections, we will describe the stepwise approach to the classification of IGPs.  Figure 1 shows these stepwise approaches. Firstly, the dataset collection and preprocessing method will be discussed. The feature representation method will be presented in the next section; the classification framework and model evaluation will be disused in the third stage of the methodology.

### 2.1. Dataset Construction and Preprocessing

This portion will discuss dataset collection for experimenting, i.e., training and evaluating the designed framework. The dataset contained the immunoglobulins sequences downloaded from the UniProt database present in or outside the cell membrane. There are some standard techniques to assure the quality of the baseline dataset; in the first stage, we eliminated the ambiguous residues, i.e., “B,” “J,” “O,” “X,” “U,” and “Z” from the protein sequences to obtained typical amino acid sequences [38]. We also eliminate the sequence if it is the portion of other proteins. We picked the protein sequences from the human, mouse, and rat categories in the second stage. We used CD-HIT software to diminish hugely indistinguishable bias in the last stage, which caused overfitting predicted results, and the cutoff value is set at 60%:

(1) $$\begin{array}{c} D=D^{+}\cup D^{-}\text{.} \end{array}$$

Our dataset *D* consists of 302 samples, with 110 positive *D*^+^ and 192 negative *D*^−^ samples of immunoglobulins for training the model:

(2) $$\begin{array}{c} indD=indD^{+}\cup indD^{-}\text{.} \end{array}$$

Our independent dataset *indD* contains 112 samples to evaluate our trained model, of which 40 are positive *indD*^+^ and 72 are negative *indD*^−^ samples. Overall, 150 positive and 264 negative samples are provided in Supplementary File S1 and Supplementary File S2, respectively.

### 2.2. Existing Feature Extraction Schemes

**Figure 2:**
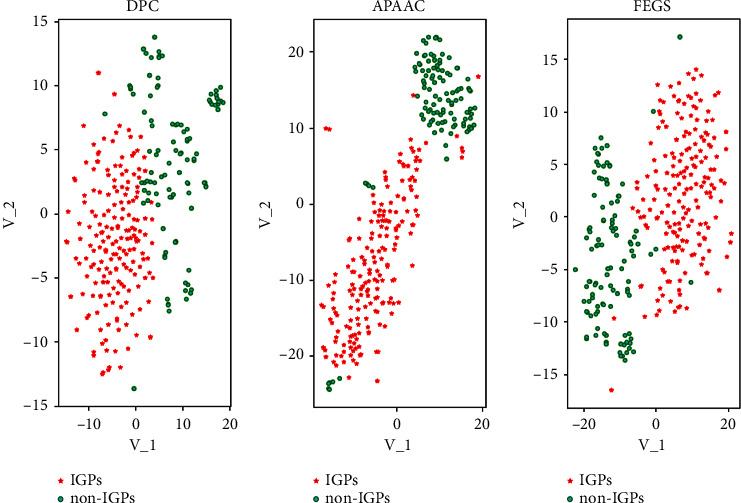
Scatter plot of DPC, APAAC, and FEGS feature extraction methods.

In designing a computerized framework, a series of steps are carried out to predict immunoglobulins. Among them, the feature extraction scheme is a challenging and essential step in formulating a biological sequence into some numerical values [39]. Conventional classification learning models, including K-nearest neighbour (KNN), random forest (RF) [40, 41], and support vector machine (SVM) [42], are based on fixed-length statistical values and are unable to handle the variable-length protein sequence; hence, the features representation algorithm can tackle this problem by extracting the fixed-length feature vector form the variable-length sequences [43–45]. Several researchers have used different feature encoding schemes [46] as shown in Figure 2; however, none of them used the proposed method for extracting vital pattern information from the immunoglobulins. A detailed description is given in Section 2.3.

### 2.3. Feature Extraction Based on Graphical and Statistical Features (FEGS)

**Figure 3:**
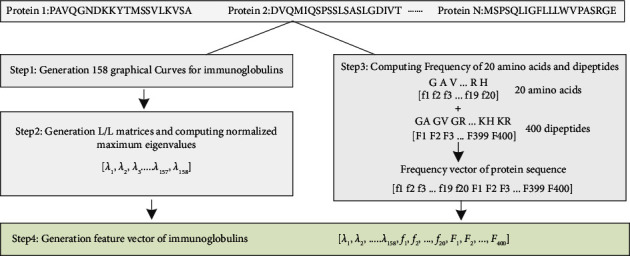
A brief explanation of the proposed FEGS.

Herein, we have opted for a novel feature representation method named Feature Extraction based on Graphical and Statistical features (FEGS) [47] for immunoglobulins sequences, as shown in Figure 3. The proposed deep neural network is not novel; however, the extraction of features through this method is novel. Extracting the hidden pattern information through graphs is different from other sequence-based feature descriptors. The main shortcoming of traditional methods is the loss of sequence order information. For example, amino acid composition and reduced amino acid alphabet cannot retain the protein's global correlated properties. Furthermore, the manual extraction of features requires extensive approaches which can be somehow not sufficient. These handcrafted features are not that much powerful to discriminate biological sequences as compared to the deep representations, as shown in [15]. The FEGS algorithm was proposed to tackle this issue by formulating the biological proteins using a three-dimensional curve. The working principle of the FEGS algorithm is that initially, FEGS employs the graphical depiction of primary proteins using circular cones in 3D space by extending the notion of 3D protein paths. Secondly, using the physicochemical properties of amino acids that efficiently extract the statistical attributes of protein pairs, FEGS seeks to form many circular cones in 3D space. Finally, the 578-dimensional vector is generated by combining mono-amino acid and dipeptide compositions for each protein sequence.

Initially, the protein sequences are provided in the FASTA format as input, and then FEGS starts eliminating unnecessary indices with identical values and generating 158 space curves for the subsequent protein sequence.

#### 2.3.1. Generation of 3D Graphical Curves for Immunoglobulins Sequences

In this method, the protein sequences are provided in the FASTA format as input; then, according to their physicochemical indices, 20 amino acids are first linked with 20 points in the 3D area. In the second step, the graphical curve of an immunoglobulins sequence can be generated by enlarging a 3D protein track centred on a right circular cone.

*(1) Preparation of the 20 Amino Acids and the 400 Amino Acid Sets*. Physicochemical properties (PCP) of amino acids (AAs) play a vital role in analyzing and characterizing protein function. We arranged the 20 AAs with respect to their PCP from lower to higher order. Then, we organized them on the circumference of the bottommost of a right circular cone with a height of 1 by the following formula:

(3) $$\begin{array}{c} \Phi \left({Α}_{i}\right)=\left(\cos\frac{2\Pi^{i}}{20},\sin\frac{2\Pi^{i}}{20},1\right),i=1,2,\ldots ,20\text{.} \end{array}$$

The above equation Α_*i*_ denoted 20 amino acids, whereas all 400 amino acid pairs are linked to the bottom of the right circular cone via the formula below:

(4) $$\begin{array}{c} \Phi \left({Α}_{i}{Α}_{j}\right)=\Phi \left({Α}_{i}\right)+\frac{1}{4}\left(\Phi \left({Α}_{j}\right)\right),i,j=1,2,\ldots ,20\text{.} \end{array}$$

Α_*i*_Α_*j*_ represents each of the 400 amino acid pairs.

*(2) Building 3D Graphical Curves for Protein Sequences*. Consider that we have a protein sequence S having N AA residues *S*=*s*_1_*s*_2_...*s*_*N*_. Constructing the 3D graph for the protein sequence is quite challenging. The 3D graphical curve is generated by enlarging a 3D protein track centred on a right circular cone as follows. Initiating from the origin point *p*_0_=(0,0,0) broadens it to the subsequent point *p*_1_(*x*_1,_*y*_1_, *z*_1_) in the 3D area, conforming to the first AA *s*_1_ and the second point *p*_2_(*x*_2_, *y*_2_, *z*_3_) related to the second AA *s*_2_ and so on till the 3D track is accomplished at the last AA *s*_*N*_, and via this process, the P path is obtained, coordinating with a 3D graphical curve of the immunoglobulins sequence S, whereas *P*_*i*_(*x*_*i*_, *y*_*i*_, *z*_*i*_) is the *i*^*th*^ amino acid *S*_*i*_, and the point coordinates *x*_*i*_, *y*_*i*_ and *z*_*i*_ are described in the following formulas:

(5) $$\begin{array}{c} \Psi \left(S_{i}\right)=\Psi \left(S_{i-1}\right)+\underset{{Α}_{1},{Α}_{2}\in \left\{A,C,D,\ldots ,Y\right\}}{\sum }f_{{Α}_{1}{Α}_{2}}.\Phi \left({Α}_{1}{Α}_{2}\right)\text{.} \end{array}$$

In the above equation, Ψ(*S*_0_)=(0,0,0), and *f*_Α_1_Α_2__ is the number of amino acid sets determined. The selected 158 physicochemical properties are linked with the exclusive right circular cone; in this way, we got 158 various 3-dimensional graphical curves for every immunoglobulin sequence related to the 158 physicochemical properties of amino acids.

#### 2.3.2. Numerical Features of Protein Sequences

Another challenging job is to transform the generated graphical curves into numerical feature vectors for the similarity analysis of immunoglobulins samples. Here, for each curve, the L/L matrix denoted by *M* is calculated, and off-diagonal values *M*_*i*,*j*_(*i* ≠ *j*) are well-defined as a measure of the Euclidean distance and the sum of geometric lengths of boundaries between *P*_*i*_ and *P*_*j*_ of the curve. At the same time, on-diagonal elements are equal to zero. Subsequently, all 158 curves are converted into 158-dimensional feature representation matrices as a graphical features representation described below:

(6) $$\begin{array}{c} V_{g}=\left[\lambda_{1},\lambda_{2},\ldots ,\lambda_{158}\right]\text{.} \end{array}$$

There are many other feature extraction techniques in which AAC and DPC are commonly utilized in protein sequence analyses. To count the frequency of AA in a given sequence, normalized by sequence length, AAC is widely used for this process to extract 20 fixed-length features as formulated below:

(7) $$\begin{array}{c} V_{a}=\left[f_{1},f_{2},\ldots ,f_{20}\right]\text{.} \end{array}$$

The above equation *f* represents the number of AA occurrences in the protein sequence. DPC also counts the number of occurrences of the 400 AA sets of the given protein sequence; and it extracts 400 fixed-length features below:

(8) $$\begin{array}{c} V_{d}=\left[f_{1},f_{2},\ldots ,f_{400}\right]\text{,} \end{array}$$

where *f* represents the number of occurrences of *j*^*th*^ AA sets, i.e., {*AA*, *AC*, *AD*, *AE*, .*YY*} in the protein sequence. The statistical features, i.e., AA *V*_*a*_ and DPC *V*_*d*_ are merged with graphical features represented *V*_*g*_ to get a 578-dimensional feature vector for the protein sequence S. In general, a dataset that contains *N* number of immunoglobulins sequences is given to FEGS, then we can get the *N* × 578 feature representation matrix, in which every row represents a feature representation vector of immunoglobulins sequences.

## 3. The Proposed Model Workflow

**Figure 4:**
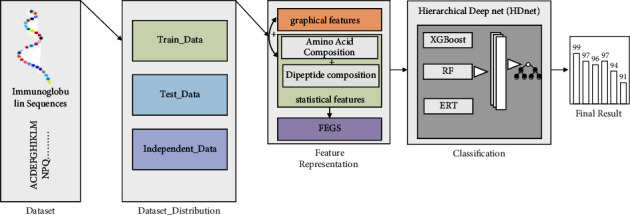
Graphical representation of the IGPred-HDnet model.

We developed a robust immunoglobulins predictor called Immunoglobulin Proteins Prediction Hierarchical Deep net (IGPred-HDnet).  Figure 4 illustrates the flow of the proposed framework, in which the main stages of the IGPred-HDnet framework are shown such as data collection, data distribution, feature representation computation through FEGS, and classification through HDNet and evaluation. In feature representation, a novel feature encoding method is proposed to extract valuable feature representation from immunoglobulin sequences.

### 3.1. Hierarchical Deep Net Model (HDnet)

**Figure 5:**
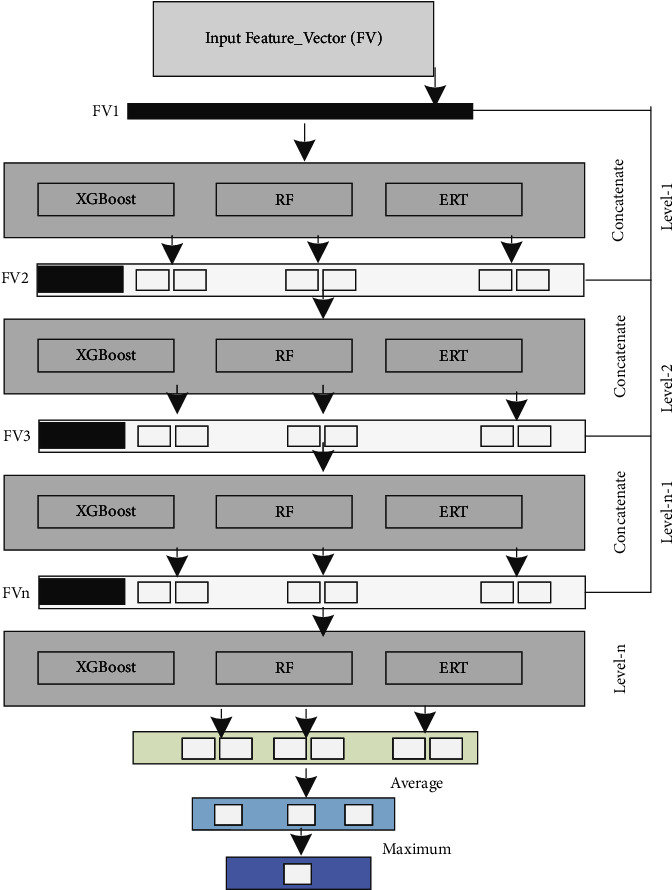
The layer-by-layer framework of the HDnet.

The hierarchical deep net (HDnet) model is an ensemble-based model inspired by [48], which is a substitute for a deep neural network (DNN) to learn hyperlevel feature representation using various resources and efforts. In contrast, DNN used complex architecture, i.e., forward and backward propagation algorithms, to learn hidden information. In developing an HDnet classifier, it is crucial to determine the learning algorithms employed in each layer. In our proposed model, we set the combination of Extreme Gradient Boost (XGBoost) [49, 50], random forest (RF) [51–53], and extremely randomized trees (ERT) [54, 55] classifiers which achieved outstanding performance and feed it with the previously computed 578-dimensional vector. HDnet is based on the deep ensemble method that cascades conventional classifiers, for example, RF, ERT, and XGBoost. Compared to DNN, HDnet uses decision trees instead of various neural network (NN) models for feature representation learning in each layer.  Figure 5 shows the generic representation of HDNet, elaborating that if there are multiple feature vectors from multiple encoding schemes, they are concatenated at the level-*N*. These feature vectors are actually deep representations learnt at different layers, similar to other deep neural networks. Due to the hierarchical type nature, the HDnet model allows the training process to be more robust, and it will be more appropriate for training a limited amount of protein samples. DNN involves various parameters that need tunes during training a model, while our proposed model easily tunes the hyperparameter.

We set the boosting parameter value *k* = 20 for the XGBoost classifier. For RF and ERT, the number of decision trees is also set at 20, and the node values are picked by randomly picking features. In our model, every layer is an ensemble of diverse learners (e.g., six XGBoost, six RF, and six ERT) who accept the feature representation processed by previous layer classification models. The outcome of the previous layer is the input for the subsequent layer for processing. To produce the enhanced feature representation related to the multivariate class vectors, we have integrated, stacked, and summed output as a supreme probability score. The process of training is terminated if enhancement is not observed in performance.  Figure 5 reveals the layer-by-layer framework of the HDnet.

## 4. Performance Evaluation

In this research, we utilized four performance evaluation measures, e.g., accuracy (ACC), specificity (SP), sensitivity (SN), and Matthew correlation coefficient (MCC), to figure out the achievement rate of our proposed prediction models described as

(9) $$\begin{array}{c} ACC=\frac{TP+TN}{TP+FP+TN+FN}\text{,}\\ SP=\frac{TN}{TN+FP}\text{,}\\ SN=\frac{TP}{TP+FN}\text{,}\\ MCC=\frac{TP*TN-FT*FN}{\sqrt{\left(TP+FP\right)*\left(TP+FN\right)*\left(TN+FP\right)*\left(TN+FN\right)}}\text{.} \end{array}$$

In the above equations, TP represents True-IGPs, which are correctly predicted as positive instances, whereas TN corresponds to true non-IGPs, which are correctly classified as negative samples. FN indicates non-IGPs, which the model incorrectly predicts as immunoglobulins.

The performance above measures containing the MCC is dependent on the threshold, which delivers the comprehensive evaluation for the binary class classification. Furthermore, to describe the model performance on a large scale, we utilized the Area Under the ROC (Receiver Operating Characteristic) Curve (AUC), which is in the shape of an independent threshold analysis like a further essential assessment of the model.

## 5. Proposed Framework Evaluation

In machine learning (ML), the model performance is naturally assessed via cross-validation (CV). There are three tests in the research community to determine the discriminatory power of the designed framework: K-fold also called subsampling, Jackknife, i.e., leave-one-out and independent tests [56, 57]. The Jackknife test provides exceptional and encouraging results to train a model [58]; however, the main cons are computational cast due to a large number of calculations [59]. To overcome the weakness of the Jackknife and improve the simplification power, we implemented the *K*-Fold CV test to train our model and test the performance [60]. In this method, we randomly divided the train data into *K-*folds (subsets), in which *K* − 1 is utilized to train the proposed model, and the leftover is utilized to test the model [61]. Subsequently, for the particular approximation, the obtained results are averaged. We set the value of *K* to 10 after conducting various experiments.

### 5.1. Predictive Performance of Hypothesis Learners Using Various Feature Encoding Schemes on Training Dataset D_train_

**Figure 6:**
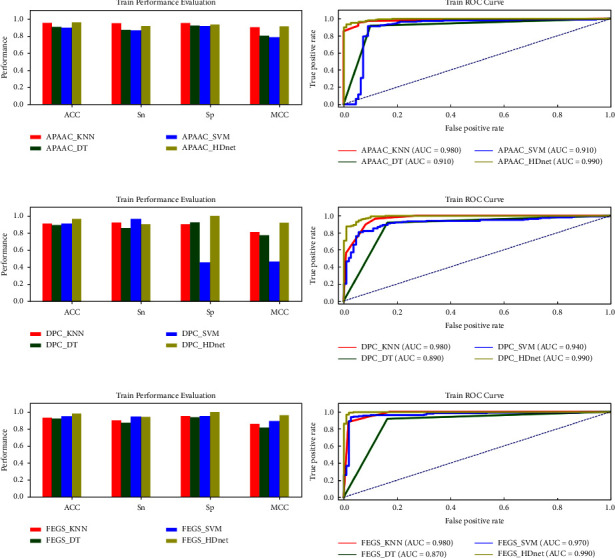
Training parameter metric performances evaluation and AUC-ROCs of KNN, DT, SVM, and the proposed HDnet via APAAC, DPC, and the proposed FEGS feature extraction methods.

In this section, we experimentally determine the prediction performance of various classifiers, i.e., KNN [62], DT [63], SVM [46, 64], and HDnet using various descriptors, i.e., APAAC (physicochemical features), DPC (sequential features), and FEGS (graphical features), as shown in Figure 6. Each learning engine is computed by conducting a ten-fold CV test on the training dataset D_train_ with four evaluation measures ACC, SN, SP, and MCC. In the case of APAAC feature vectors, the SVM classifier secured the worst AAC = 89.72% and MCC = 0.786, while HDnet achieved a higher ACC of 95.69% and MCC of 0.909 points. Similarly, in the case of the DPC method, again the HDnet classifier produced 0.33% high ACC and 0.007 points MCC, respectively. Furthermore, in the case of the FEGS feature method, the highest performance is obtained by the HDnet classifier, which is ACC = 98.00%, SN = 94.55%, SP = 100%, and MCC = 0.958. The second-best predictor is KNN which achieved 90.41% ACC and 0.809 points MCC, while SVM comparatively produce good predictions on all feature methods.

Several judgements are made on the reported results of all classifiers in Table 1. First, the HDnet model consistently produced the best outcomes among the classification algorithms compared to other machine-learning classifiers for all feature encoding schemes. The main reason is due to the high learning potential of a deep neural network as compared to the conventional classifiers. The internal structure of the HDnet classifier is based on decision trees that enable the model to predict the extracted features better [65]. Further, it is evident in the literature that deeper networks have more learning potential as compared to conventional neural networks [15, 66, 67].

Secondly, among the feature representation approaches, FEGS (graphical features) produced the best results for overall hypothesis learners (classifiers) than other feature vectors such as DPC and APAAC. The underlyingreason for the high prediction rate of FEGS methods is that FEGS extracts the conserved local and global graphical, physicochemical and statistical attributes from a protein sequence. As in Figure 1, the visualization influence of the extracted features through t-distributed stochastic neighbour embedding (t-SNE) can be seen. The red colour represents the IGPs class, and the green colour represents the non-IGPs class. The features with a high correlation, like DPC and APAAC, cannot incorporate the correct predictions of immunoglobulins. In contrast, the novel features of FEGS are less correlated enabling the classifiers to produce high performance.

### 5.2. Predictive Performance of Hypothesis Learners Using Various Feature Encoding Schemes on the Testing Dataset D_test_

**Figure 7:**
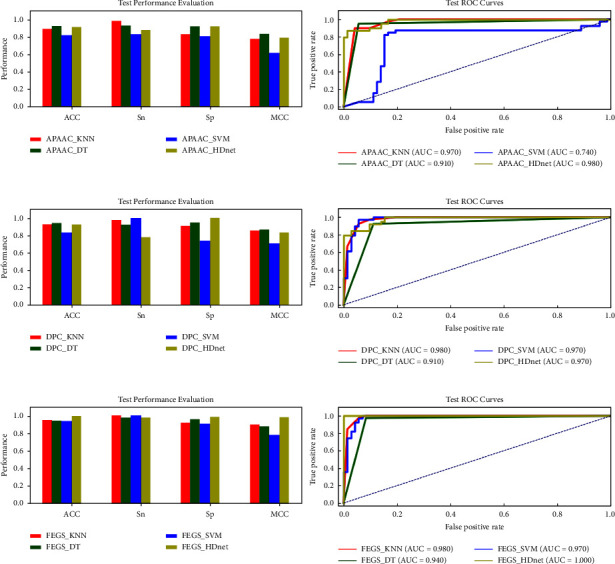
Training parameter metric performances evaluation and AUC-ROCs of KNN, DT, SVM, and the proposed HDnet via APAAC, DPC, and the proposed FEGS feature extraction methods.

In this subsection, we examine the success rates of our model via an independent test to show its generalization power. It was ensured that the samples in the independent test D_test_ were unseen, and none of the immunoglobulin samples was used in training the model. Table 1 depicts the prediction outcomes of all classifiers using the APAAC, DPC, and FEGS feature methods. Comparative analysis reveals that our proposed learning model HDnet using novel feature FEGS achieved outstanding results in terms of all performance metrics, likewise ACC = 99.10%, SN = 97.50%, SP = 100%, and MCC = 0.980 points, respectively. In contrast, the same learning engine using the APAAC feature produced the worst results as shown in Figure 7.

### 5.3. Predictive Performance of the Proposed Predictor with Existing Methods on Training and Testing Datasets

**Figure 8:**
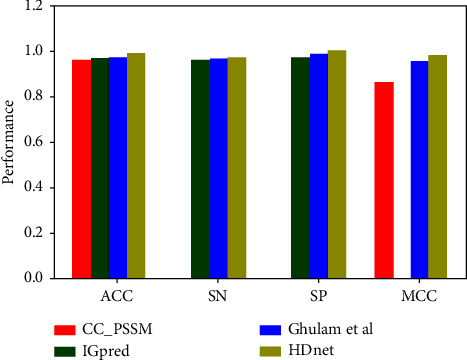
Comparison of the proposed method with the existing methods.

In this section, we theoretically compare the efficacy of our proposed model with the three developed approaches such as CC-PSSM [39], IGPred [19], and Ghulam et al.'s approach [68] on training and testing datasets. The results in Table 2 are extracted from the previous literature [69]. It is worth noting that none of the existing predictors generated the prediction outcomes on independent tests to show the generalization power of their model. Driven by the novel feature descriptor FEGS with the intelligent deep learning-based algorithm HDnet, IGPred-HDnet outperformed the existing methods for IGs identification in terms of all performance metrics, i.e., ACC, SN, SP, MCC, and AUC. On the training benchmark dataset, our method notable increased ACC by 1.9%, SN by 1%, SP by 1.5%, and MCC by 0.026 points over the second-best performer XGBoost. An independent test was performed to investigate further the IGPred-HDnet model's predictive capability on unseen data. Both the ACC and AUC results are 0.99 and 1.00, as shown in Table 2 and Figure 8.

The underlying reason for achieving high predictions is to extract the graphical-based, physicochemical-based, and sequence-based attributes. Also, the hierarchical type structure of the HDnet classifier enables a better forecast of the IGs samples from the extracted attributes [65].

## 6. Conclusion and Future Work

IGPs are a crucial constituent of the immune system. Understanding deep insight IGPs can provide useful hints in drug discovery for disease treatment. Thus, the objective of this research was to construct a novel sequence-based computational method for predicting and analyzing IGPs. The proposed theoretical model “IGPred-HDnet” is superior to other advance immunoglobulin-based predictors due to several reasons. Firstly, we designed an innovative graphical algorithm FEGS to capture structured information buried in the protein sample. The structure features produced better results than the other feature schemes. Secondly, we implemented a deep learning model called HDnet for the first time as a learning model for recognizing IGPs.

Despite enhancing the model's overall performance, further gaps still exist for future, such as several previous publications like Tang et al. [27] established public webservers that can enrich the applicability of the anticipated model. Also, using novel feature selection algorithms is vital to avoid overfitting and improve the generalization power of the trained model. We hope that the proposed IGPred-HDnet will become a potential tool for large-scale IGPs characterization in particular and other protein problems in general.

## Figures and Tables

**Table 1:** Analysis of various classifiers using feature encoding schemes on training and testing datasets D_train_ and D_test_.

Feature-encoding methods	Classifiers	Benchmark dataset					Independent dataset				
		ACC (%)	SN (%)	SP (%)	MCC	*F*-measure (%)	ACC (%)	SN (%)	SP (%)	MCC	*F*-measure (%)
**APAAC**	KNN	95.01	94.55	95.26	0.898	92.95	88.93	97.50	83.33	0.777	85.71
	DT	90.70	87.27	92.66	0.802	86.92	91.96	92.50	91.66	0.829	89.15
	SVM	89.72	86.36	91.66	0.786	85.61	81.25	82.50	80.55	0.612	75.86
	HDnet	**95.69**	**91.82**	**93.75**	**0.909**	**93.83**	**90.17**	**87.50**	**91.66**	**0.787**	**86.41**

**DPC**	KNN	90.41	91.82	89.63	0.809	86.77	92.85	97.50	90.27	0.854	90.69
	DT	89.39	85.45	91.66	0.771	85.11	93.75	92.05	94.44	0.864	91.35
	SVM	90.23	96.36	45.58	0.467	69.67	83.03	100	73.61	0.706	80.80
	HDnet	**96.02**	**90.00**	**99.47**	**0.916**	**94.18**	**91.96**	**77.50**	**100**	**0.829**	**87.32**

**FEGS**	KNN	93.03	90.00	94.78	0.856	89.71	94.64	100	91.60	0.890	93.00
	DT	91.93	87.27	93.74	0.817	87.95	93.75	97.50	91.60	0.871	91.76
	SVM	94.72	94.55	94.85	0.889	92.96	93.75	100	90.27	0.876	91.95
	HDnet	**98.00**	**94.55**	**100**	**0.958**	**96.94**	**99.10**	**97.50**	**100**	**0.980**	**98.73**

**Table 2:** Analysis of various classifiers using feature encoding schemes on training and testing datasets D_train_ and D_test_.

Dataset	Predictor	ACC	SN	SP	MCC	Pre	NPV	F1	AUC
Training	CC_PSSM	0.960	—	—	0.921	0.961	—	—	0.994
	IGPred	0.969	0.963	0.975	—	—	—	—	0.994
	XGBoost	0.972	0.945	0.985	0.950	0.980	—	—	0.970
	IGPred-HDnet	**0.980**	**0.945**	**1.000**	**0.958**	**1.000**	**1.000**	**0.971**	**0.998**

Testing	CC_PSSM	0.883	—	—	0.847	0.884	—	—	0.914
	IGPred	0.891	0.886	0.897	—	—	—	—	0.914
	XGBoost	0.894	0.869	0.906	0.874	0.902	—	—	0.892
	IGPred-HDnet	**0.991**	**1.000**	**0.986**	**0.980**	**0.9750**	**1.000**	**0.987**	**1.000**

## Data Availability

The dataset analyzed in this study can be found in the supplementary files.

## Supplementary Materials

Supplementary Materials Supplementary File S1 contains the positive samples (immunoglobulins sequences). Supplementary File S2 contains the negative samples (nonimmunoglobulins sequences).
